# Red meat and dietary iron intakes are associated with some components of metabolic syndrome: Tehran Lipid and Glucose Study

**DOI:** 10.1186/s12967-019-2059-0

**Published:** 2019-09-18

**Authors:** Zohre Esfandiar, Firoozeh Hosseini-Esfahani, Parvin Mirmiran, Ali-Siamak Habibi-Moeini, Fereidoun Azizi

**Affiliations:** 1 Nutrition and Endocrine Research Center, Research Institute for Endocrine Sciences, Shahid Beheshti University of Medical Sciences, Tehran, Iran; 2 Endocrine Research Center, Research Institute for Endocrine Sciences, Shahid Beheshti University of Medical Sciences, Tehran, Iran

**Keywords:** Red meat, Dietary iron intake, Metabolic syndrome

## Abstract

**Background:**

This study was conducted to investigate whether the daily consumption of haem, non-haem, total iron and red meat can affect the occurrence of metabolic syndrome (MetS) and its components.

**Methods:**

Eligible adults (n = 4654) were selected from among participants of the Tehran Lipid and Glucose Study with an average follow-up of 3.8 years. Dietary intakes were assessed using a valid and reliable semi-quantitative food frequency questionnaire. Anthropometrics and biochemical variables were evaluated at baseline and follow-up examinations. The occurrence of MetS and its components were assessed in relation to haem, non-haem, total iron and red meat intakes.

**Results:**

There was no relationship between different types of dietary iron and red meat intakes and the incidence of MetS in the Tehranian population. Risk of hypertension decreased from quartiles 1 to 4 for haem iron (HR: 1.00, 0.92, 0.81, 0.80, P_trend_ < 0.01) and red meat intake (HR: 1.00, 0.89, 0.84, 0.77, P_trend_ < 0.01). The association between hyperglycemia and the fourth quartile of total iron intake was significant (HR = 1.98, 95% CI 1.08–3.63); and the risk of high triglyceride appeared to increase in higher quartiles of total iron intake (HR: 1.00, 1.17, 1.49, 1.75, P_trend_ = 0.01) compared to lower quartiles.

**Conclusion:**

Our study suggests a potentially protective relationship of haem and moderate red meat intake against development of high blood pressure; and higher intake of total iron is related to hyperglycemia and high triglyceride.

## Background

The prevalence of metabolic syndrome (MetS) has increased in older ages and 25% of adults suffer from MetS [1]. MetS is a cluster of metabolic abnormalities including hyperglycemia, dyslipidemia, high blood pressure (BP) and central obesity, which increases the risk of type 2 diabetes [2], cardiovascular disease [3], specific cancers [4] and mortality [5]. The close relationship between MetS and diet has been approved [6] and there is concern on finding which nutrients or foods reduce or increase the risk of MetS.

The results of previous studies indicate that meat consumption (especially red meat) is associated with an increased risk of MetS, which may be related to the high level of iron and saturated fat in meat [2, 7–9]. Some studies have shown an association between the ferritin levels in serum and MetS [10–12]. Iron overload is specified by an increment in the serum ferritin levels [13], and some studies have reported that meat or heme iron intake is related to the serum ferritin [14, 15]. A few studies have demonstrated the association between consumption of dietary iron and MetS, which could potentially address the causative character of the association between iron metabolic markers and MetS [16–18]. Iron is present in foods in a heme or non-heme form, which present differences in absorption, bioavailability, metabolism and food sources. Heme iron is more efficiently absorbed than non-heme iron as nearly 25% heme iron and 5% non-heme iron from diet absorbed by intestine [19]. The Iranian diet is known to be plant-based, which implies a low bioavailability of dietary iron because of high contribution of non-haem iron in diet [20].

As far as we know, there is no study about the association between dietary iron and MetS in the Middle east; hence, the aim of the current study was to determine the association of haem, non-haem, total iron and red meat intakes with MetS and its components in an Iranian population.

## Methods

### Study population

Subjects of this cohort study were selected from participants of the Tehran Lipid and Glucose Study (TLGS), a population-based prospective study performed to determine the risk factors for non-communicable diseases in a sample of residents from District 13 of Tehran, the capital of Iran (20). The first examination survey was performed from 1999 to 2001 on 15,005 individuals aged ≥ 3 years, using the multistage stratified cluster random sampling technique, and follow-up examinations were conducted every 3 years; 2002–2005 (survey 2), 2005–2008 (survey 3), 2008–2011 (survey 4), and 2012–2015 (survey 5) to identify recently developed diseases.

**Fig. 1:**
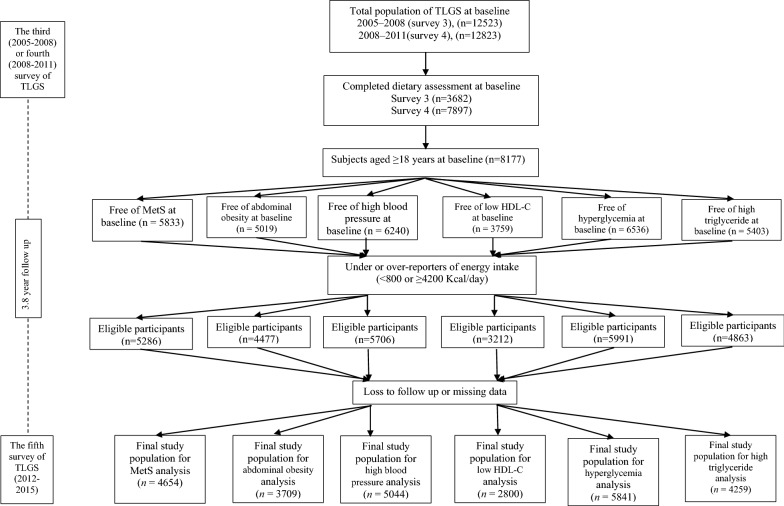
Outline of study participants’ selection

Of individuals participating in surveys 3 and 4, respectively 3682 and 7897 subjects were randomly selected for dietary assessment. For the current study, a total of 8177 adult men and women aged ≥ 18 years with available dietary, biochemical and anthropometric data were selected as the baseline population and followed until survey 5 (participants entered at surveys 3 and 4 had respectively followed two times and one time for the outcome measurements). Of these participants, we excluded pregnant or lactating women, those with under- or over-report of energy intake (< 800 or ≥ 4200 kcal/day) (n = 547) and also subjects with prevalent MetS (n = 2325) at baseline. Finally, after excluding participants missing any follow up data (n = 632), 4654 subjects remained and entered the analysis. Other separate lines of exclusion were performed for components of metabolic syndrome, including high triglyceride, low high density lipoprotein cholesterol (HDL-C), abdominal obesity, high fasting blood sugar (FBS) and high BP (Fig. 1).

All participants signed a written informed consent form before taking part in this investigation. The study was implemented based on the Declaration of Helsinki and the study protocol was accepted by the ethics committee of the Research Institute for Endocrine Sciences, Shahid Beheshti University of Medical Sciences, Tehran, Iran. All methods were performed in line with their relevant guidelines and regulations.

### Dietary intake measurements

Dietary assessment was performed by a valid and reliable 168-item semi-quantitative food frequency questionnaire (FFQ); expert dietitians collected information on the intake of standard serving sizes of a list of foods, through face-to-face personal interviews. The consumption frequency of each food item on a daily, weekly, or monthly basis was converted to daily intakes; portion sizes were then converted to mass (in grams), using household measures. Since the Iranian food composition table (FCT) is incomplete, the United States Department of Agriculture (USDA) FCT was used to analyze foods [21]. Red meat was defined as the sum of beef, lamb, organ meats (kidney, beef liver and heart) and processed meats (sausages and hamburger). Haem iron was estimated as 40% of the total iron from poultry, fish, beef, lamb, organ meats and processed meats [22]. Non-haem iron was calculated as the difference between total iron and haem iron.

### Physical activity

Physical activity level was evaluated using the Persian-translated modifiable activity questionnaire with high reliability and relative validity. Information on the time and frequency of light, moderate, high, and very high severity activities were collected according to the list of usual activities of daily life over the past year. Physical activity levels were reported based on the metabolic equivalent-h/week (Met/h/week) [23–25].

### Anthropometric measurements

Weight was measured to the nearest 100 g, using digital scales (Seca 707), while the subjects were minimally clothed and not wearing shoes. Height was measured to the nearest 0.5 cm by a tape measure, in standing position with shoulders in normal alignment and without shoes. Waist circumference (WC) was taken at the end of normal expiration, over light clothing, with a non-flexible tape meter at the level of the umbilicus without any pressure to body surface; measurements were recorded to the nearest 0.1 cm.

### Laboratory assays

Blood samples were drawn into vacutainer tubes from subjects who were in sitting position between 7:00 to 9:00 a.m., after a 12–14 h overnight fast. Blood samples were centrifuged within 30 to 45 min of collection. All biochemical analyses were performed using a Selectra 2 auto-analyzer at the TLGS research laboratory on the day of blood collection. FBS concentration was measured by the enzymatic colorimetric method using the glucose oxidase technique. HDL-C concentration was assessed after precipitation of the apolipoprotein B-containing lipoproteins with phosphotungstic acid. triglyceride level was determined by enzymatic colorimetric tests using glycerol phosphate oxidase and triglyceride kits. Assay performance was monitored once in every 20 tests, using lipid control serum, Percinorm (normal range) and Percipath (pathological range), where applicable (Boehringer Mannheim; catalog no. 1446070 for Percinorm and 171778 for Percipath). A lipid standard (Cfas, Boehringer Mannheim; catalog no. 759350) was used to calibrate the Selectra 2 auto-analyzer on each day of the laboratory analysis, and all samples were analyzed only when the internal quality control met the standard criteria. Inter- and intra-assay coefficients of variations were both 2.2% for serum glucose and 1.6% and 0.6% for triglyceride, respectively [26].

### Definitions

Individuals with three or more of the following criteria for MetS were considered as unhealthy phenotypes based on the Iranian modified National Cholesterol Education Program/Adult [27, 28]: (1) Abdominal obesity (WC ≥ 95 cm in men and women); (2) BP ≥ 130/85 mmHg or antihypertensive drug treatment; (3) HDL-C < 1.30 mmol/l (< 50 mg/dl) in women, and < 1.04 mmol/l (< 40 mg/dl) in men or receiving drug treatment; (4) FBS ≥ 6.11 mmol/l (≥ 110 mg/dl) or drug treatment for hyperglycemia; (5) triglyceride ≥ 1.70 mmol/l (≥ 150 mg/dl) or drug treatment.

### Statistical analyses

Statistical analyses were carried out using the Statistical Package for Social Sciences (version 21.0; SPSS). A two-tailed P value < 0.05 was used to determine statistical significance. All types of iron and red meat intakes were adjusted for total energy intake using the residual model [29]. We used a Chi square test for qualitative variables and the one way ANOVA for quantitative variables to compare the characteristics across quartiles of the average energy-adjusted daily intake of total iron. In case of non-normal nutritional and biochemical variables (triglyceride concentration), log-transformed values were used for statistical analysis. The hazards ratio (HR) and 95% confidence interval of incident MetS and its components were assessed using multivariable Cox proportional hazard regression models. The incidence of MetS or its components during the follow up period were considered as dichotomous variables (yes/no) in the models. Different types of dietary total iron (Q1 < 13.87, Q2: 13.87–16.03, Q3: 16.04–19.85, Q4 > 19.85 mg/day), haem (Q1 < 0.26, Q2: 0.27–0.39, Q3: 0.40–0.57, Q4 > 0.57 mg/day), non-haem (Q1 < 13.45, Q2: 13.46–15.51, Q3: 15.52–19.17, Q4 > 19.17 mg/day) iron and red meat (Q1 < 30.50, Q2: 30.51–36.33, Q3: 36.44–49.91, Q4 > 49.91 g/day) intake were categorized into quartiles, given the first quartile as the reference. The survival time for censored individuals was calculated as the interval between the first and last observation dates. The event date for the incidence of MetS and its components was considered as the mid-time between the date of the follow up visit at which the events were diagnosed for the first time, and the most recent follow up visit preceding the diagnosis. Study participants were censored due to loss to follow-up, the end of the observation period or death. The median of each quartile was used as a continuous variable to assess the overall trends of HRs across quartiles of dietary iron and red meat intakes in the Cox proportional hazard regression models. The proportional hazard assumption of multivariate Cox models were assessed using Schoenfeld’s global test of residuals.

The confounders were selected based on literature, also each confounder was included in the univariable Cox regression model. A two-tailed P value < 0.20 was used for determining inclusion in the model. The Cox regression models were adjusted for several potential confounders; the analyses were adjusted for sex, age, BMI, education levels (> 14 and ≤ 14 years), smoking (never smoked, past smoked, and current smoker), physical activity (continuous), dietary iron supplements, fiber (gr/1000 kcal), saturated fat (percentage of energy), magnesium, vitamin C and total energy intake; in models for estimating HR of high BP and high triglyceride, sodium (continuous) and total fat (percentage of energy) have been added, respectively. In Cox regression models where haem iron was a predictive variable, non-haem iron was included in the model as an adjustment variable and vice versa.

## Results

### Characteristics of the participants

General characteristics of the study population across quartiles of total dietary iron intake are presented in Table 1. Subjects in the lower quartiles of total iron intake were younger; also, they had a higher percentage of smokers and lower BMI. Dietary intakes including carbohydrate, protein, fiber, vitamin C and magnesium were significantly different among quartiles of total iron intake and also the prevalence of MetS and its components were significantly different among quartiles of total iron intake.

**Table 1:** Baseline characteristics of the study population, across the quartiles of total iron intake in the Tehran Lipid and Glucose Study

Characteristic	Total iron consumption (mg/day)				
	Q1	Q2	Q3	Q4	P
Baseline age (years)	38.19 ± 14.1^a^	41.54 ± 14.4	42.30 ± 14.6	40.42 ± 14.1	< 0.01
Women, % (n)	27.4 (887)	24.0 (778)	22.3 (722)	26.4 (856)	< 0.01
Current smokers (%)	24.6	21.6	23.5	20.0	< 0.01
Education level (%)†	22.5	27.4	30.1	32.7	< 0.01
Physical activity (MET/min/week)	584 ± 891	536 ± 837	553 ± 805	546 ± 834	0.34
BMI (Kg/m^2^)	25.2 ± 4.9	25.9 ± 4.8	25.8 ± 4.8	25.7 ± 4.9	< 0.01
Energy intake (kcal/day)	2440 ± 715	2076 ± 664	2346 ± 724	2530 ± 698	< 0.01
Carbohydrate (% of energy)	53.8 ± 6.7	58.6 ± 5.7	60.5 ± 6.5	60.5 ± 10.5	< 0.01
Protein (% of energy)	13.5 ± 2.2	14.4 ± 2.7	14.9 ± 3.9	15.0 ± 9.4	< 0.01
SFA (% of energy)	12.1 ± 5.2	10.0 ± 2.3	9.1 ± 2.9	10.0 ± 21.4	< 0.01
Fiber (g/1000 kcal)	13.5 ± 4.5	18.0 ± 6.2	21.5 ± 8.9	20.2 ± 11.0	< 0.01
Vitamin C (mg/day)	59.8 ± 32.5	67.3 ± 35.0	74.8 ± 42.0	81.1 ± 50.0	< 0.01
Magnesium (mg/day)	159 ± 30	181 ± 34	198 ± 43	202 ± 44	< 0.01
Total iron (mg/day)	12.2 ± 4.0	14.9 ± 4.7	18.8 ± 5.9	23.6 ± 7.9	< 0.01
Haem iron (mg/day)	0.49 ± 0.29	0.53 ± 0.32	0.51 ± 0.34	0.50 ± 0.30	< 0.01
Non-Haem iron (mg/day)	10.7 ± 1.7	13.2 ± 1.3	14.7 ± 2.2	15.8 ± 3.4	< 0.01
Red meat (g/day)	30.5 ± 19.6	32.6 ± 18.4	31.9 ± 20.4	31.1 ± 18.0	< 0.01
Metabolic syndrome (%)	19.7	26.0	28.0	26.1	< 0.01
Abdominal obesity (%)	34.0	41.0	43.8	40.5	< 0.01
High blood pressure (%)	20.1	25.5	27.9	25.6	< 0.01
Low HDL-C (%)	62.7	59.5	56.7	51.2	< 0.01
Hyperglycemia (%)	14.3	20.6	24.5	25.5	< 0.01
High triglyceride (%)	31.3	38.3	39.2	34.9	< 0.01

*Q* quartiles of total iron consumption, *MET* metabolic equivalent, *BMI* body mass index, *MUFA* mono-unsaturated fatty acids, *PUFA* poly-unsaturated fatty acids, *SFA* saturated fat

^a^Values are mean  ±  SD unless otherwise listed

^†^Educational level ≥ 14 years

### Association of dietary iron intake with MetS

After an average follow-up of 3.8 years, new-onset of MetS was developed in 1106 participants. The association between MetS development and the quartiles of iron (total, haem and non-haem) and red meat intake are presented in Table 2. In the crude model, subjects in the upper quartile of total and non-haem iron intake had a higher risk of incident MetS than those in the lowest quartile (P_trend_ < 0.05). High consumption of red meat was associated with a lower MetS risk (P_trend_ < 0.01); However, when potential confounders were considered, the statistical significance of crude models disappeared.

**Table 2:** Hazard ratios (95% CI) of metabolic syndrome across energy-adjusted quartiles of iron (total, haem and non-haem) and red meat intake in adult participants of the Tehran Lipid and Glucose Study

Characteristic	Quartiles of dietary iron and red meat intake				
	Q1	Q2	Q3	Q4	P_trend_
Total iron (mg/day)	< 13.87	13.87–16.03	16.04–19.85	> 19.85	
Crude	1.00 ref.	1.14 (0.97–1.34)	1.40 (1.19–1.65)	1.18 (0.98–1.43)	0.02
Model 1^a^	1.00 ref.	0.97 (0.79–1.19)	1.10 (0.81–1.49)	2.04 (0.97–4.28)	0.22
Haem iron (mg/day)	< 0.26	0.27–0.39	0.40–0.57	> 0.57	
Crude	1.00 ref.	0.94 (0.80–1.11)	0.85 (0.72–1.01)	0.84 (0.71–1.00)	0.35
Model 1^b^	1.00 ref.	0.90 (0.71–1.14)	0.89 (0.70–1.12)	0.87 (0.67–1.12)	0.30
Non-Haem iron (mg/day)	< 13.45	13.46–15.51	15.52–19.17	> 19.17	
Crude	1.00 ref.	1.10 (0.93–1.30)	1.36 (1.15–1.61)	1.44 (1.21–1.71)	< 0.01
Model 1^c^	1.00 ref.	0.98 (0.78–1.24)	1.16 (0.89–1.52)	1.15 (0.80–1.63)	0.46
Red meat (g/day)	< 30.50	30.51–36.33	36.44–49.91	> 49.91	
Crude	1.00 ref.	0.83 (0.70–0.99)	0.85 (0.72–1.01)	0.69 (0.58–0.82)	< 0.01
Model 1^a^	1.00 ref.	0.86 (0.55–1.26)	0.96 (0.68–1.28)	0.87 (0.56–1.24)	0.43

^a^Adjusted for age, sex, baseline BMI, educational level, smoking status, total energy intake, fiber, saturated fat, sodium, vitamin C and magnesium intakes

^b^Adjusted for age, sex, baseline BMI, educational level, smoking status, total energy intake, fiber, saturated fat, sodium, vitamin C, magnesium, and non-haem iron intakes

^c^Adjusted for age, sex, baseline BMI, educational level, smoking status, total energy intake, fiber, saturated fat, sodium, vitamin C, magnesium and haem iron intakes

### Association of dietary iron intake with components of MetS

HR and 95% confidence interval of the MetS components for energy-adjusted quartiles of iron and red meat intakes are shown in Table 3.

**Table 3:** Hazard ratios (95% CI) of metabolic syndrome components across energy-adjusted quartiles of iron (total, haem and non-haem) and red meat intake in adult participants of the Tehran Lipid and Glucose Study (n=7630)

Characteristic	Quartiles of dietary iron and red meat intake				
	Q1	Q2	Q3	Q4	P_trend_
Total iron					
Abdominal obesity^a^	1.00 ref.	0.74 (0.54–0.95)	1.18 (0.79–1.76)	1.11 (0.34–2.32)	0.68
High blood pressure^b^	1.00 ref.	1.00 (0.90–1.13)	1.13 (0.97–1.37)	1.04 (0.59–1.84)	0.52
Low HDL-C^a^	1.00 ref.	1.02 (0.72–1.44)	1.20 (0.73–1.99)	1.38 (0.31–5.99)	0.07
Hyperglycaemia^a^	1.00 ref.	0.98 (0.81–1.18)	1.07 (0.81–1.42)	1.98 (1.08–3.63)	0.19
High triglyceride^c^	1.00 ref.	1.22 (0.96–1.56)	1.62 (1.14–2.29)	1.89 (0.80–4.04)	0.01
Haem iron (mg/day)					
Abdominal obesity^d^	1.00 ref.	0.82 (0.59–1.14)	0.74 (0.52–1.04)	0.88 (0.62–1.25)	0.44
High blood pressure^e^	1.00 ref.	0.93 (0.81–1.07)	0.82 (0.71–0.95)	0.81 (0.70–0.94)	0.00
Low HDL-C^d^	1.00 ref.	0.67 (0.44–1.01)	0.67 (0.44–1.02)	0.93 (0.61–1.42)	0.99
Hyperglycaemia^d^	1.00 ref.	0.96 (0.77–1.20)	1.04 (0.83–1.30)	0.96 (0.75–1.22)	0.80
High triglyceride^f^	1.00 ref.	0.98 (0.75–1.30)	1.14 (0.87–1.51)	1.16 (0.87–1.55)	0.19
Non-Haem iron					
Abdominal obesity^g^	1.00 ref.	0.73 (0.54–1.00)	0.90 (0.52–1.28)	0.96 (0.60–1.54)	0.58
High blood pressure^h^	1.00 ref.	1.03 (0.92–1.15)	1.13 (0.99–1.31)	1.19 (0.98–1.44)	0.32
Low HDL-C^g^	1.00 ref.	1.01 (0.79–1.28)	1.13 (0.89–1.43)	1.21 (0.95–1.53)	0.64
Hyperglycaemia^g^	1.00 ref.	0.98 (0.79–1.21)	1.03 (0.80–1.33)	1.24 (0.90–1.71)	0.21
High triglyceride^i^	1.00 ref.	1.00 (0.75–1.34)	1.09 (0.95–1.75)	1.48 (0.99–2.74)	0.05
Red meat					
Abdominal obesity^a^	1.00 ref.	0.75 (0.55–1.03)	0.74 (0.53–1.04)	0.82 (0.59–1.14)	0.47
High blood pressure^b^	1.00 ref.	0.90 (0.79–1.02)	0.82 (0.71–0.96)	0.76 (0.65–0.87)	0.00
Low HDL-C^a^	1.00 ref.	0.96 (0.76–1.21)	1.14 (0.91–1.42)	0.95 (0.76–1.19)	0.85
Hyperglycaemia^a^	1.00 ref.	1.03 (0.82–1.30)	1.00 (0.80–1.24)	0.91 (0.72–1.13)	0.23
High triglyceride^c^	1.00 ref.	1.09 (0.81–1.43)	1.07 (0.82–1.48)	1.11 (0.82–1.45)	0.42

^a^Adjusted for age, sex, baseline BMI, educational level, smoking status, total energy intake, fiber, saturated fat, vitamin C and magnesium intakes

^b^Additionally adjusted for sodium

^c^Additionally adjusted for total fat

^d^Additionally adjusted for non-haem iron

^e^Additionally adjusted for sodium and non-haem iron

^f^Additionally adjusted for total fat and non-haem iron

^g^Additionally adjusted for and haem iron

^h^Additionally adjusted for sodium and haem iron

^i^Additionally adjusted for total fat and haem iron

Risk of hypertension decreased from quartiles 1 to 4 for haem iron (HR (95% CI) 1.00, 0.93 (0.81, 1.07), 0.82 (0.71, 0.95), 0.81 (0.70, 0.94), P_trend_ < 0.01) and red meat intake (HR (95% CI) 1.00, 0.90 (0.79, 1.02), 0.82 (0.71, 0.96), 0.76 (0.65, 0.87), P_trend_ < 0.01).

With respect to quartile one, participants in the fourth quartile had a higher risk of hyperglycemia (HR = 1.98, 95% CI 1.08–3.63); and the risk of high triglyceride appeared to increase significantly in higher quartiles of total iron intake (HR (95% CI) 1.00, 1.22 (0.96, 1.56), 1.62 (1.14, 2.29), 1.89 (0.80, 4.04), P_trend_ = 0.01) compared with the lower quartiles.

## Discussion

The current investigation was a prospective cohort study, evaluating the association of dietary iron and red meat intakes with MetS or its components. Our results suggested that there was no relationship between any type of dietary iron and red meat intake, and the incidence of MetS in the Tehranian population. Incidence of hypertension decreased with high intake of haem iron and red meat intake, after adjusting for several confounders, and total iron intake was positively associated with hyperglycemia and high triglyceride.

### Red meat, dietary iron intakes and MetS

This non-significant association between MetS and red meat intake was similar to previous results in the Asian population but not Western population. Consistent with our study, a recent meta-analysis study showed an inverse but non-significant association between MetS and red meat intake [RR = 0.91 (95% CI 0.82, 1.00)] in the Asian population. However, the Western population had a 33% higher risk of MetS in the upper category of red meat intake compared to those in the lowest intake category. This discrepancy in the effect of red meat intake may be substantially due to lower consumption of red meat in our study population (total meat intake in the highest quartile was less than 2 serving/day) and also, in the Asian population compared to the Western [9, 30]. According to the OECD (Organization for Economic Cooperation and Development), consumption of meat is significantly low in Asian countries [31].

In line with our study, no association was reported between haem iron intake and the risk of MetS in a recent cross-sectional study in people Republic of China [17]. This result was inconsistent with studies on Western populations [16, 32], which showed that intake of haem iron is associated with MetS. The amount of haem iron intake might explain this inconsistency. Compared to haem iron intake of the Western population, the average intake of heam iron in the current study was lower (haem median dietary iron intake was 0.39 mg/day) [16, 32–34]. Heam iron is a stronger predictor of serum ferritin compared to non-haem iron. Elevated serum ferritin levels have appeared as a characteristic in individuals with MetS [10, 34].

### Red meat, dietary iron intakes and components of MetS

A negative association was observed between high BP, and haem iron and red meat intake. Kim et al. [35] reported that Korean children and adolescents who consumed more than 5 servings of red meat and chicken per week, had a lower prevalence of high blood pressure compared to those who consumed less than 5.

The BOLD Study showed that systolic blood pressure reduced after the intake of DASH (Dietary Approaches to Stop Hypertension) diet with 153 g of lean beef (main source of haem iron) per day, but did not reduce after DASH diets including 113 g or 28 g of lean beef per day [36]. It seems that moderate red meat intake decreased high blood pressure. In addition, subjects classified into total and non-haem iron consumption quartiles showed an ascending trend for intake of fiber from quartiles 1 to 4 for total and non-haem iron. Increasing fiber intake can improve BP [37].

A positive relationship has been shown between total iron intake and incident hyperglycemia, in two cross-sectional and one prospective study [16, 38, 39]. Iron overload can act as a strong pro-oxidant, and cause oxidative stress and damage to tissues such as pancreatic beta cells, which can decrease the synthesis and secretion of insulin, impair insulin signaling, and finally, change glucose metabolism [40, 41]. A direct association between iron intake and the increased risk of hyperglycemia has been previously found [16, 42].

The mechanisms underlying the associations between total iron intake and high triglyceride levels are uncertain. Although, we hypothesize that high intake of iron may lead to increased risk of iron overload and then enhance the generation of inflammation, which can cause insulin resistance and then hyperinsulinaemia; these conditions may reduce insulin-mediated suppression of hormone-sensitive lipase (enzyme responsible for mobilization of triglyceride), which can increase intracellular lipolysis, plasma levels of free fatty acids and their transport to the liver. The increment in the levels of liver free fatty acids motivates triglyceride-rich lipoprotein production [43–48]. Also, our results are along with previous reports showing a relationship between red meat (the main source of iron) intake and high triglyceride levels [8, 16, 45]. In our population non-haem iron had a higher contribution of total iron; as well there was a borderline association between non- haem iron and high triglyceride (P_trend_ = 0.05). main source of non-haem in our population is grains (specially refined grains) [20], which reveals that higher intake of non- haem iron is associated with higher intake of simple carbohydrates. It can explain the reason of hyperglycemia [49] and high triglyceride [50].

The present study has important strengths. The comparison of associations with total, haem and non-haem iron provided insight into the role of these different types of iron in the incidence of MetS and its components. The prospective design allowed the estimation of incident disease with less worry about reverse causality between nutrients and outcomes. The evaluation of nutrient consumption from various food sources provided a new vision into the association between disease and nutrients. Limitations, include lack of data on serum levels of iron and total iron binding capacity, and under or over estimations of dietary intakes as an inherent limitation of FFQ. Despite that the nutrients were adjusted for important confounders, some confounders such as CRP were not included.

## Conclusion

Our study suggests that a higher consumption of haem iron red meat is negatively associated with elevated blood pressure, and a high intake of total iron is related to hyperglycemia and high triglyceride. Furthermore, the present study stresses the important role of moderate red meat intake on blood pressure.

## Data Availability

The datasets used and/or analyzed during the current study are available from the corresponding author on reasonable request.

## Abbreviations

MetS: metabolic syndrome
BP: blood pressure
TLGS: Tehran Lipid and Glucose Study
HDL-C: high density lipoprotein cholesterol
FFQ: food frequency questionnaire
FCT: food composition table
USDA: United States Department of Agriculture
Met: metabolic equivalent
WC: waist circumference
FBS: fasting blood sugar
MUFA: mono unsaturated fatty acids
PUFA: poly unsaturated fatty acids
SFA: saturated fatty acids
